# Metabolic reconstruction of the archaeon methanogen *Methanosarcina Acetivorans*

**DOI:** 10.1186/1752-0509-5-28

**Published:** 2011-02-15

**Authors:** Vinay Satish Kumar, James G Ferry, Costas D Maranas

**Affiliations:** 1 Joint BioEnergy Institute, 5885 Hollis street, Emeryville, California 94608, USA; 2 Department of Biochemistry and Molecular Biology, The Pennsylvania State University, University Park, PA 16802, USA; 3 Department of Chemical Engineering, The Pennsylvania State University, University Park, PA 16802, USA

## Abstract

**Background:**

Methanogens are ancient organisms that are key players in the carbon cycle accounting for about one billion tones of biological methane produced annually. *Methanosarcina acetivorans*, with a genome size of ~5.7 mb, is the largest sequenced archaeon methanogen and unique amongst the methanogens in its biochemical characteristics. By following a systematic workflow we reconstruct a genome-scale metabolic model for *M. acetivorans*. This process relies on previously developed computational tools developed in our group to correct growth prediction inconsistencies with *in vivo *data sets and rectify topological inconsistencies in the model.

**Results:**

The generated model *i*VS941 accounts for 941 genes, 705 reactions and 708 metabolites. The model achieves 93.3% prediction agreement with *in vivo *growth data across different substrates and multiple gene deletions. The model also correctly recapitulates metabolic pathway usage patterns of *M. acetivorans *such as the indispensability of flux through methanogenesis for growth on acetate and methanol and the unique biochemical characteristics under growth on carbon monoxide.

**Conclusions:**

Based on the size of the genome-scale metabolic reconstruction and extent of validated predictions this model represents the most comprehensive up-to-date effort to catalogue methanogenic metabolism. The reconstructed model is available in spreadsheet and SBML formats to enable dissemination.

## Background

Genome-scale metabolic models (for recent reviews, see [1] and [2]]) are increasingly becoming available for an expanding range of organisms. There exists at least forty completed bacterial and thirteen eukaryotic metabolic reconstructions with many more under development [1]. In the past decade, several studies [3] have demonstrated a variety of uses ranging from strain optimization [4-6] pathogen drug target identification [7,8], bacterial community metabolic interactions [9] and identification of human disease biomarkers [10]. In contrast to the extensive interest devoted towards bacterial and eukaryotic metabolism reconstruction, efforts to construct archaeal metabolic models have been noticeably limited [11,12]. This is partly due to the current relative paucity of -omics datasets available for species from the *Archaea *domain. This dearth of data, however, is likely to change in the near future as recent interest in methanogenic *archaea *has led to several sequencing efforts [13-15], as well as transcriptomic and proteomic analyses [16-20]. Furthermore, it is increasingly becoming apparent that archaeal metabolism has significant implications to the earth's climate [21] thus motivating the need to globally assess their true metabolic capabilities by reconstructing high quality metabolic models.

Methanogens (def., methane-producing) constitute the largest group of the *Archaea *domain of life [22]. Methanogens are terminal organisms in anaerobic microbial food chains (i.e., consortia) that break down organic matter to methane in diverse anaerobic environments in a process that helps regulate the global carbon flux [23]. The process plays a surprisingly significant role in global warming accounting for about one billion tons of the annual methane production [21,24]. Recently, Cheng and coworkers used a consortia of methanogens to convert electricity into methane thereby paving the way for converting electric current generated by renewable energy sources into renewable biofuels [25]. On the evolutionary front, methanogens are amongst the most ancient form of life on earth and their role as the progenitors of the first eukaryotic cell has been suggested under two separate hypotheses [26,27]. In addition, unique biochemical properties such as broad substrate specificity, participation of novel coenzymes in the methanogenesis pathways and the presence of unique lipids in their cell wall set these organisms apart from the bacterial and eukaryotic branches of life [28]. Therefore, the reconstruction of archaeal methanogen metabolic models could help paint a more complete picture of life's metabolic processes.

Feist and coworkers first developed a genome-scale model (named *i*AF692) [11] for the fresh-water methanogen, *Methanosarcina barkeri *using a draft version of its genome. In this paper, we reconstruct a genome-scale metabolic model for the marine methanogen, *Methanosarcina acetivorans. M. acetivorans *is an aceticlastic methanogen that was first isolated from marine microbial communities that degrade kelp into methane [29]. At over 5.7 million base pairs [15], it has the largest reported genome of all sequenced *Archaea *(about 20% larger than the *M. barkeri *genome) alluding to an expanded metabolic repertoire. This repertoire includes unique methanogenic pathways, broader substrate specificity than other methanogens and a large number of duplicate genes [15,18-20,30,31]. Recent studies have shown that *M. acetivorans *uniquely exhibits both methanogenic and acetotrophic growth on carbon monoxide [31]. All these unique metabolic characteristics and planet-wide carbon balance impact [21,24] provide the motivation to globally assess its metabolic capabilities.

Draft metabolic reconstructions generated using homology-based comparisons unavoidably contain some omissions and misclassifications. These errors are manifested either as unreachable metabolites or as *in silico *predictions that are in contrast with observed *in vivo *behavior [32,33]. In response to these challenges, Suthers et al., proposed a computational workflow to generate and curate the metabolic models and applied it to the metabolic reconstruction of *Mycoplasma genitalium *[34]. The proposed workflow makes use of two separate model correction procedures. GapFind and GapFill identify and subsequently restore connectivity to unreachable metabolites [33] and GrowMatch that reconciles *in silico *growth predictions with *in vivo *growth data [32]. In this paper, we streamline this workflow for the generation of an archaeal metabolic model and customize it to the available types of data.

We first generated a draft reconstruction of *M. acetivorans *using homology comparisons with the published model [11] of the fresh-water methanogen, *M. barkeri*. We then deployed a modified version of the workflow presented in Suthers et al., by combining the GapFind/GapFill and GrowMatch steps of the procedure [34]. The completed model accounts for 941 genes, 705 reactions and 708 metabolites. The model also predicted substrate specific phenotypes of *M. acetivorans *and captured unique bioenergetics exhibited by the organism across different conditions.

## Results and Discussion

The metabolic model reconstruction workflow consists of four steps. Step 1 refers to the draft model generation using bidirectional blast (BBH) and database/literature searches. Step 2 involves model modifications to ensure biomass formation for growth under all known substrates. Step 3 applies GrowMatch [32] to restore growth prediction inconsistencies and Step 4 applies GapFind and GapFill [33] to restore connectivity.

### Step 1: Generating Draft model

BBH searches for each of the 692 genes included in the *i*AF692 model were conducted against the latest genome sequence of *Methanosarcina acetivorans C2A *strain [15]. At this stage of the reconstruction process, we included only open reading frames (ORFs) that have e-values (in both directions) of at most 10^-30^. This process yields an initial conservative model for *M. acetivorans *that has 776 genes. Based on the primary TIGR annotation of *M. acetivorans *[35] this accounts for 17.07% (776/4540) of all ORFs in the *M. acetivorans *genome. The mapping of the metabolic genotypes between these two very closely related organisms is surprisingly complex. Specifically, 369 one-to-one mappings, 1,113 one-to-many mappings and 1,050 many-to-many mappings (*M. barkeri *to *M. acetivorans*) were generated. The large number of one-to-many and many-to-many mappings is consistent with the incidence of a high number of gene duplicates in the *M. acetivorans *genome (539 multigene families) and accounts for the additional 84 genes in *i*VS941 over *i*AF692 [15].

We use multiple sources to annotate the remaining 3,764 ORFs in the genome. Specifically, we *preferentially *assigned metabolic annotation to seven genes based on the information available at an organism-specific annotation effort for *M. acetivorans *[36], 51 genes based on SEED annotations [37] and 107 genes based on TIGR annotations. Interestingly out of these 165 genes as many as 68 code for isozymes. Predicted or hypothetical proteins account for the remaining 2,411 ORFs not included in the model after the annotation step. Approximately 44% of all genes in *M. acetivorans *(upon excluding hypotheticals and predicted proteins) were present in the draft metabolic model. The methanogenesis pathways in the *M. acetivorans *model were modified to account for known differences documented in the literature.

Specifically, *M. acetivorans *and *M. barkeri *use different electron transport chains to generate ATP when they grow on acetate. The electron transport chain in *M. barkeri *consists of a pair of hydrogenases, Ech and Vho that couple hydrogen production/oxidation to proton translocation outside the membrane [30]. In *M. acetivorans*, ECH and VHO are absent and instead it is hypothesized that an electron transfer complex Rnf (abbreviation in *i*VS941: RNF) oxidizes reduced ferredoxin to generate a sodium ion gradient which is then exchanged for a proton gradient by the multiple resistance/pH regulation Na+/H+ antiporter (abbreviation in *i*VS941: MRP) [30]. *M. acetivorans *grows on carbon monoxide as a substrate in the absence of hydrogen using both the electron transport chain (the methanogenic (methane forming) pathway) and substrate level phosphorylation (acetogenic (acetate forming) pathway). Alternatively, it has been proposed that *M. barkeri *grows on CO only in the *presence *of hydrogen and oxidizes CO to CO_2 _and uses the resulting energy to produce hydrogen that is then reoxidized using the hydrogenases (discussed above) to produce electrons needed to reduce CO to methane [30]. On C1 compounds such as methanol and methylamines, both organisms have a methylotrophic pathway that disproportionates the carbon to form carbon dioxide and methane [38]. Interestingly, one mole of substrate is oxidized to generate reducing equivalents required to produce three moles of methane.

In contrast with other archaeal models [11,12], we delineated methyltransferase specificity [39,40] for different substrates of *M. acetivorans *. We also generated the Gene-Protein-Reaction mappings for the *M. acetivorans *model using as a starting point the *i*AF692 model based on the AUTOGRAPH method developed by Notebaard and coworkers [41]. All exchange reactions and non-gene associated intracellular reactions available in the *i*AF692 model were also appended to the model, as we did not find any evidence to the contrary [see Methods]. Upon conclusion of Step 1, a draft model with 941 genes, 705 reactions and 708 metabolites was generated.

### Step 2: Model correction to enable biomass formation

We determine the metabolic capabilities of the assembled draft model to grow on known methanogenic substrates by first specifying the biomass equation and then specifying the composition of the minimal medium. The first requirement is addressed by assuming that the set of components that compose the biomass equation in *M. acetivorans *is identical to the one used in the *i*AF692 model. The stoichiometric coefficients of the nucleotide components (datp, dgtp, dctp, dttp, ctp and gtp) were modified to reflect the difference in the G/C contents of the two organisms (see Additional File 1). The utilization of the same biomass component set is supported by experimental data on the minimal medium (Ferry et al., unpublished data). The minimal growth medium contained six additional vitamins and trace elements (pyridoxine-HCL, sodium molybdate, thioctic acid, nitrilo tri acetic acid and boric acid) over the one used in *i*AF692 [11]. We chose to exclude them from our model as no metabolic role for them was identified based on literature searches or gleaned by the model.

Equipped with the biomass composition and the minimal medium, we tested the capability of the draft model to enable growth on the following known methanogenic substrates: carbon monoxide, acetate, methanol and monomethylamine, dimethylamine and trimethylamine [29]. The draft model did not exhibit growth on any of these substrates motivating the use of GapFind [33] to identify the biomass precursor metabolites that could not be produced using these substrates in a minimal medium. GapFind revealed that the same precursor metabolite Adenosylcobalamin-HBI could not be produced for all substrate choices in the draft model. We used GapFill [33] to restore flow through this metabolite. This was achieved under all aforementioned substrate conditions by adding an exporter for the cofactor, tetrahydrosarcinapterin. While the export of the cofactor could be an *in silico *response to an imbalance of cofactors and there is no evidence in the literature for the presence of a tetrahydrosarcinapterin exporter, it is possible that an enzyme outside the cell wall may utilize the cofactor as a substrate.

### Step 3: Evaluating and improving model performance using GrowMatch

After ensuring *in silico *growth on a defined medium across different substrates, we further examined the model by testing for growth prediction agreement with experimental data across different genetic/environmental perturbations. Using literature searches, we assembled a dataset consisting of *in vivo *growth data for 60 different conditions (See Table 1). As shown in Table 1, growth data was assembled for eighteen genetic perturbations for growth on methanol, thirteen on acetate as carbon substrates, nine on carbon monoxide as carbon and energy source, and 20 on methylamines as carbon substrates. Not surprisingly, most of these gene deletions are in methanogenesis pathways (Table 1) indicative of the significant attention this pathway has received before.

**Table 1:** *In vivo *gene deletion data used evaluate and improve *i*VS941's predictive capabilities (Citations are indicated in square brackets).

	Substrate					
Gene deletions	acetate	carbon monoxide	methanol	monomethylamine	dimethylamine	trimethylamine
ackR	NGNG	**GNG**[51]	-	-	-	-
ATP synthase	NGNG[52]	-	**GNG**[52]	-	-	-
DMTsD	GG[53]	GG[53]	GG[53]	-	-	GG[53]
mtsD+mtsF	GG[53]	GG[53]	GG[53]	-	-	GG[53]
mtsD+mtsH	GG[53]	GG[53]	GG[53]	-	-	GG[53]
mtsF	GG[53]	GG[53]	GG[53]	-	-	GG[53]
mtsH	GG[53]	GG[53]	GG[53]	-	-	GG[53]
mtsF+mtsH	GG[53]	GG[53]	GG[53]	-	-	GG[53]
lysK	-	-	GG[54]	-	GG[54]	GG[54]
lysS	-	-	GG[54]	GG[54]	GG[54]	GG[53]
MCR	NGNG[43]	**GNG**[43]	NGNG[43]	**GNG**[43]	**GNG**[43]	**GNG**[43]
mtaA1	-	-	NGNG[39]		-	-
mtaA1 + MT1	**GNG**[39]	-	-	-	-	-
mtaA2	-	-	GG[39]	-	-	-
mtaCB1	-	-	GG[55]	-	-	-
mtaCB1 + mtaCB2	-	-	GG[55]	-	-	-
mtaCB1 + mtaCB2 + mtaCB3	-	-	**GNG**[55]	-	-	-
mtaCB2	-	-	GG[55]	-	-	-
mtaCB3	-	-	GG[55]	-	-	-
mtbA	-	-	-	NGNG[39]	NGNG[39]	-
mtbA	-	-	-	-	-	GG[54]
ppylT	GG[56]	-	GG[56]	**GNG**[56]	**GNG**[56]	**GNG**[56]
ptaR	NGNG	**GNG**[51]	-	-	-	-
Rnf complex	**GNG**[57]	-	-	-	-	-

In line with previous approaches [42] the growth cutoff for classifying a mutant as a "Growth" or a "No-Growth" mutant was chosen to be 1/3^rd ^of average growth across the dataset. Using this cutoff and the terminology introduced in the GrowMatch procedure [32] we arrive at 43 GG (mutant exhibits *in silico *and *in vivo "***G**rowth") thirteen GNG (mutant exhibits *in silico *"**G**rowth" and *in vivo *"**N**o-**G**rowth") and eight NGNG (mutant exhibits *in silico *and *in vivo *"**N**o-**G**rowth") cases. Notably, the incidence of only GNG model/experimental discrepancies indicates that the draft model tends to over-predict the metabolic capabilities of the organism when in error. A closer examination reveals that in 31 out of 43 GG cases the deleted genes correspond to isozymes while six correspond to deletions of methyltransferases. In all these cases both the model and the experiment agree that the deleted genes are non-essential. Of the nine GNG cases that could be resolved, eight were resolved by conditionally suppressing one additional reaction and one was resolved by carrying out a single global suppression (global suppressions do not affect any consistent GG cases when carried out globally whereas conditional suppressions affect atleast one GG case)(see Additional File 1).

**Figure 1:**
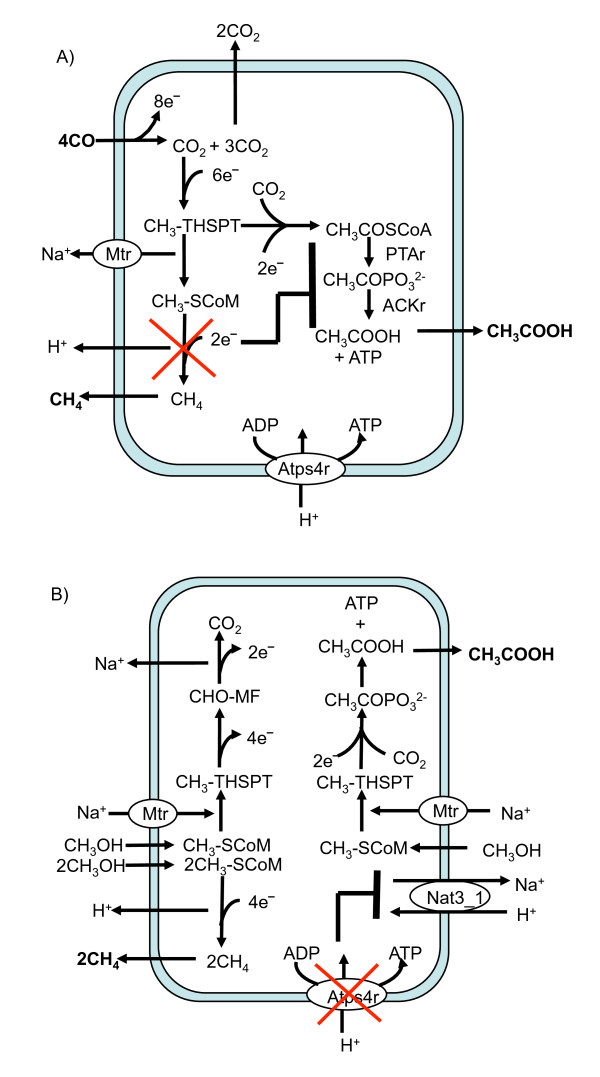
**Resolving GNG mutants using GrowMatch**. A) GrowMatch resolution of the GNG mutant characterized by deleting Methyl Coenzyme Reductase with carbon monoxide as the carbon source. B) GrowMatch's resolution of the GNG mutant characterized by deleting ATP Synthase with methanol as the carbon source

Figure 1 highlights two examples of GrowMatch's resolution of GNG mutants in *M. acetivorans*. As shown in Figure 1(A), the genes encoding for Methyl-coenzyme M reductase (MCR) (the reaction that forms methane) under growth on Carbon Monoxide are non-essential *in silico *and essential *in vivo *[43]. GrowMatch suggests suppressing either the reaction catalyzed by acetate kinase (ACKr) or phosphotransacetylase (PTAr) to restore consistency to this mutant. These hypotheses are consistent with the bioenergetics when *M. acetivorans *grows on CO as the sole energy source [31]. Both the acetogenic and methanogenic branches of the methanogenesis pathway are energy yielding. Flux in the methanogenic branch results in a proton and sodium ion gradient which is then used to synthesize ATP catalyzed by the proton translocating ATP synthase. Alternatively, flux through the acetogenic branch results in ATP synthesis using substrate level phosphorylation when acetyl phosphate is converted to acetate by acetate kinase. When Methyl-coenzyme M reductase is deleted there is no mechanism to recycle HS-CoM for another round of methylation and the Mtr-catalyzed methyl transfer reaction (methyl-THSPT:CoM-SH methyltransferase) coupled to generation of the sodium gradient is also deactivated thereby diverting methyl tetrahydrosarcinapterin (CH_3_-THSPT) towards synthesis of acetate and ATP. Therefore suppressing ACKr (or equivalently PTAr) in a mutant lacking Methyl coenzyme reductase (and consequently, the methane forming branched pathway) ensures that both energy yielding pathways are deactivated thereby halting growth.

In the second case (Figure 1(B)), deleting ATP synthase results in a GNG mutant when the organism grows on methanol as the sole carbon and energy source [19]. This deletion negates proton-coupled generation of energy *via *methanogenesis leaving substrate level generation of energy *via *acetogenesis. GrowMatch suggests restoring consistency to this mutant by suppressing the sodium proton antiporter (abbreviation in *i*VS941: Nat3_1). Suppressing this reaction in this mutant metabolic network deactivates flux in the sodium-dependent reaction methyl-THSPT:coenzyme M methyltransferase (abbreviation in *i*VS941: MTSPCMMT) which results in no flux in the acetogenesis pathway (Figure 1B)).

### Step 4: Network connectivity analysis and restoration

After evaluating and improving the model using *in vivo *gene deletion data, we used the automated procedures GapFind and GapFill [33] to identify and rectify any remaining network connectivity inconsistencies. Using GapFind, we identify 92 metabolites (i.e., 13.1% of all metabolites in model) that cannot be produced for any choice of carbon substrate. Not surprisingly, none of the 95 no production metabolites were present in the methanogenesis pathway alluding to the completeness of its reconstruction. Interestingly, of the 161 metabolites present in the *M. acetivorans *model but absent in *i*AF692, 101 can be produced whereas 60 have blocked production routes. Notably, GapFind revealed that 35 out of these 95 metabolites could also not be produced in the *i*AF692 model of *M. barkeri. *Flow restoration to all 95 metabolites was attempted using GapFill by adding reactions from KEGG [44]. In this step, we restored consistency to only two of the 92 no production metabolites. Flow through these metabolites was restored by treating two existing reactions (cob(I)alamin-HBI adenosyltransferase and hydroxyethylthiazole kinase) as reversible.

### Model characteristics for *i*VS941

Table 2 contrasts the model statistics for the *i*VS941 model against previously constructed archaeal models. Most reaction entries in *i*VS941 model are associated with very stringent e-values implying a high confidence for their inclusion. Furthermore, the inclusion of seven regulatory constraints that allow for substrate specific activation of methyltransferases and the addition of reactions using multiple pieces of evidence are unique features of this model. Finally, in contrast to the remaining models, the *i*VS941 model documents global and conditional suppressions based on systematic evaluation of model predictions with *in vivo *growth data and network gap correction.

**Table 2:** Comparison between *i*VS941 and other available *Archaeal *models

	*Methanosarcina acetivorans*	*Methanosarcina barkeri*	*Halobacterium salinarum*	*Methanococcus jannaschii*
**Genome size**	5.7 Mb	4.8 Mb	2.7 Mb	1.7 Mb
**ORF's**	4540	3680	2867	1792
**Metabolic genes**	941	692	490	436
**Unique proteins**	941	542	490	266
**Reactions**	705	619	708	609
gene-associated	590	509	568	297
non gene-associated	115	110	133	312
transport reactions		88	111	1
**Metabolites**	708	558	557	510
**Gaps**	93	35		
**Consistency with growth data**	93.3%	69%	-	-

**Figure 2:**
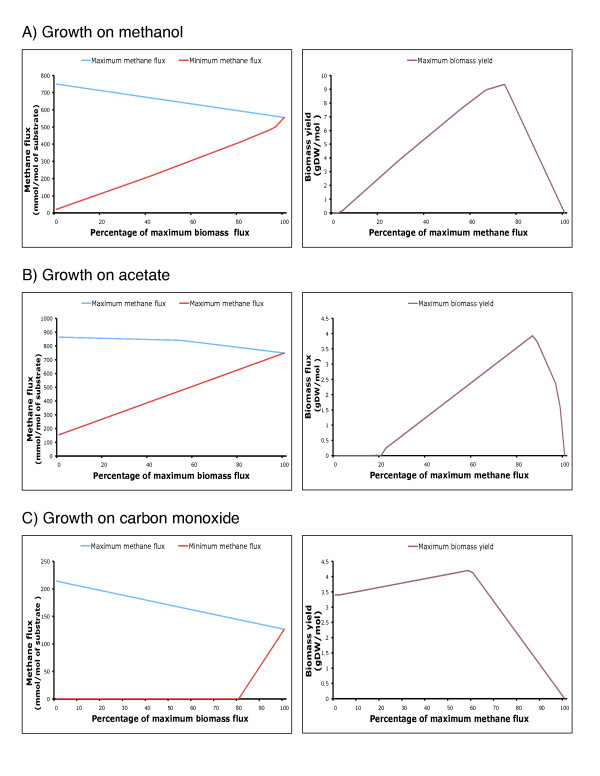
**Flux coupling analysis between yield in Methyl coenzyme reductase and biomas yield on A) methanol, B) acetate, C) carbon monoxide**. All values of yields in mmol/gDW hr^-1 ^and are normalized to the respective substrate uptake rates fixed at 1000 mmol/gDW hr.

We compared flux values through the methane forming reaction catalyzed by Methyl Coenzyme Reductase and the biomass equation to ascertain the extent of coupling between flux in the methanogenesis pathway and *in silico *growth rates. We identified the range of methane production flux by maximizing and minimizing flux through the MCR reaction for different values of biomass formation. Conversely, we identified the range of biomass production for different values of required methane production. Figure 2 shows these plots for growth on methanol, acetate and carbon monoxide.

As shown in Figure 2(A) and 2(B), a positive biomass flux implies a non-zero MCR flux for growth in methanol and acetate but not the reverse. Using the terminology introduced in [45], this implies that the flux in biomass reaction is *directionally coupled *to the flux in MCR. This is consistent with the indispensability of the methanogenic branch when *M. acetivorans *grows on acetate and methanol [30,43]. Moreover, the maximum biomass formation is reached at when the flux through MCR is fixed at 74% of its maximum value for growth on methanol and 86% for growth on methanol. At maximum biomass production, the ratio of biomass to methane production is 0.016 GDW/mmol and 0.005 GDW/mmol for growth on methanol and acetate, respectively. This higher biomass yield is qualitatively consistent with the higher energetic yield per mole of methanol observed for *M. acetivorans *[46].

Figure 2(C) illustrates the predictions of the *i*VS941 model for growth on carbon monoxide as the sole carbon and energy substrate. The model prediction that the methanogenic branch is dispensable when *M. acetivorans *grows on carbon monoxide is consistent with the mechanism proposed in [26,31]. The proposed mechanism hypothesizes alternate means of ATP generation (electron transport chain: methanogenic branch or substrate level phosphorylation: acetogenic branch) when *M. acetivorans *grows on carbon monoxide. Notably, the maximum biomass production is achieved at 58% of the maximum flux in the MCR reaction and the ratio of the two fluxes is 0.033 GDW/mmol. It has been previously established that the acetogenic and methanogenic branches of the pathway are energy yielding when *M. acetivorans *grows on carbon monoxide [31]. Using the coupling analysis described above, we find that the acetogenic and methanogenic branches are *not *coupled This supports the independence of the energy yielding branches for growth on carbon monoxide.

## Conclusions

Metabolic reconstruction technology has been used extensively to document the metabolic repertoire of organisms in the *Bacteria *and the *Eukarya *domains [47]. Here, we take advantage of the increased availability of experimental datasets and genomic information for archaeal organisms to build the metabolic model, called *i*VS941, of the archaeon with the largest known genome, *Methanosarcina acetivorans*. The *i*VS941 model is constructed using a systematic procedure that enables sequential evaluation and improvement of model capabilities. The model consists of 705 reactions, 708 metabolites and 941 genes; the latter accounting for about 44% of all ORFs in *M. acetivorans *with putative annotations [35]. The completed model has metabolites (87%) that can be produced and it has a high agreement of 93.3% against published *in vivo *growth data across environmental and genetic perturbations (thirty data points) with specificity of 81% (i.e., percent of correctly identified essential genes) and selectivity of 89.7% (i.e., percent of correctly identified non-essential genes). Additionally, the model recapitulates substrate-specific energetic characteristics such as ATP synthase indispensability for growth on acetate/methanol and its dispensability for growth on carbon monoxide.

The number of reactions included in the draft model under Step 1 is quite sensitive to the adopted BLAST cutoff. The number of gene entries increases to 1,090 when the cutoff is relaxed to 10^-20 ^from the 776 entries for the adopted cutoff of 10^-30^. This more stringent cutoff was chosen to ensure that the draft model did not contain any falsely added functionalities. We have found that it is much easier to find and add missing functionalities than correctly identifying and removing erroneous ones. Interestingly, all but one reaction in the methanogenesis pathway known to occur in *M. acetivorans *were included in the draft model using the most stringent cutoff. Reaction ECH Hydrogenase which is known to occur in *M. barkeri *but not in *M. acetivorans *was excluded from the draft model.

This constructed *i*VS941 model represents the most comprehensive up-to-date effort to catalogue methanogenic metabolism. Given the attention methanogenic consortia have received and the growing amount of metagenomic data [48], this model can be used to assess the biological impact on carbon balance of methanogenic communities. This organism-specific compilation of the metabolic repertoire of *M. acetivorans *can serve as the framework for fusing additional experimental data on methanogens as they become available. The model is available in SBML format to enable dissemination (Additional File 2).

## Methods

### Generation of initial model

We generate the initial model for *M. acetivorans *by taking advantage of an existing genome-scale metabolic model for the marine methanogen *M. barkeri *(*i*AF692). The *i*AF692 model is based on a draft version the *M. barkeri fusaro *genome [11]. We first mapped the genes from *i*AF692 onto the current genome-sequence of *M. barkeri *to restore consistency with the most up-to-date genomic information. For every gene in the *i*AF692 model, we retrieved the corresponding protein sequence (personal communication with Adam Feist of UCSD) and conducted bidirectional BLAST (BBH) (BLASTp [49]) searches against the current genome sequence of *M. barkeri*.

The draft reconstruction for *M. acetivorans *is generated by conducting bidirectional BLAST (BLASTp) searches for each one of the 692 genes in *i*AF692 against its genome and including only those genes/protein/reaction associations with an e-value of better than 10^-30^. We used multiple sources to annotate the remaining genes in *M. acetivorans *in the following order. The primary resource was an ongoing effort at the University of Maryland (carried out in the Sowers Lab at the Center for Marine Biotechnology). Whenever such information was lacking we alternatively relied on first the SEED database [37] and finally the TIGR [35] annotations.

Upon obtaining annotations for the remaining genes, we pinpointed metabolic genes by searching each annotation against the KEGG ligand [44] database and retrieving corresponding reactions. KEGG reactions are not necessarily charge/mass balanced. We manually checked the reactions we added and found that reactions involving tRNA molecules were not mass balanced. For annotations with no synonyms in the KEGG ligand database, we use their Enzyme Commission Number (if available) to search the Swiss-Prot database [50] and retrieve the metabolic reaction(s) (if at all) they are associated with. Finally, we also included reactions that are known to be present in *M. acetivorans *but absent in *M. barkeri *(e.g., reactions for CO metabolism. We use the AUTOGRAPH procedure developed by Notebaard et al., to generate the gene-protein-reaction (GPR) associations [41]. This procedure uses bidirectional BLAST hits (BBH) to generate GPR's for new metabolic reconstructions (*M. acetivorans *in our case) using the GPR's of related metabolic models (*M. barkeri*). We also added non-gene associated reactions and exchange reactions in *i*AF692 to the model unless we found evidence to contrary.

### Model fidelity improvement using available data sources

Upon the generation of the draft model the next step involves the systematic elimination of network gaps using GapFind/GapFill [33] and growth prediction inconsistencies using GrowMatch [32]. These procedures are deployed in a synergistic manner to provide mutually corroborating evidence for model correction.

#### Step 1

We generate the draft model as discussed above.

#### Step 2

We test the ability of the model to grow on known substrates. If it doesnt, we use modified versions of GapFind and GapFIll respectively to identify biomass precursors that cannot be produced and ensure their production. We allow for addition of functionalities at this step only if the BLAST e-value is lower than 10^-2^. Upon completion of this step all biomass components are available in *i*VS941.

#### Step 3

We compare *in silico *biomass production in *i*VS941 against available *in vivo *growth data across different environmental/genetic perturbations. Mutants are classified as Grow/Grow (GG), No-Grow/Grow (NGG), Grow/No-Grow (GNG) and No-Grow/No-Grow (NGNG) following the definitions proposed in [32]. GNG mutants are resolved by identifying global/conditional suppressions in the *i*VS941 network using GrowMatch. Upon completion of this step, all *in silico/in vivo *growth inconsistencies that could be corrected by either removing or adding reactions available in databases resolved.

#### Step 4

We next identified metabolites that cannot be produced or consumed using GapFind. Using GapFill, we restore connectivity to them by appending only reactions that have BBH e-values of less than 10^-10^.

In addition, we mined for all published articles having the word "Acetivorans" anywhere in their content in the Web of Science and PubMed databases and download these articles using EndNote^Web^. We used the mdfind command on a MacBook™, search for articles that have loci-names of *M. acetivorans *genes included in the *i*VS941 Model. This enables a relatively quick search for literature evidence supporting (or not) annotations in the *i*VS941 Model. We update the model to resolve any incorrect annotations identified in this step and consolidate information from articles not included in the above search domain but have information regarding methanogenesis [39].

## Authors' contributions

CDM conceived the study. VSK built the model and performed the computational analyses. VSK, JGF and CDM drafted the manuscript. All authors read and approved the final version of the manuscript.

## Supplementary Material

Additional file 1 **The *i*VS941 model in spreadsheet format**. Click here for file

Additional file 2 **The *i*VS941 model in SBML format**. Click here for file
